# Rehabilitation of stage-one scapholunate instability (ReSOS): An online survey of UK practice

**DOI:** 10.1177/17589983241268056

**Published:** 2024-08-16

**Authors:** Martin K Holmes, Caroline Miller, Michael Mansfield

**Affiliations:** 1 School of Sport, Exercise and Rehabilitation Sciences, 1724 University of Birmingham, Birmingham, UK; 2 Sandwell & West Birmingham NHS Foundation Trust, Birmingham, UK; 3 University Hospitals Birmingham NHS Foundation Trust, Birmingham, UK; 4 Clinical Academic Lead Nurses, AHPs and Midwives, Deputy Clinical Director of Research, Clinical Specialist Physiotherapist Upper Limb, University Hospitals Birmingham NHS Foundation Trust, Birmingham, UK; 5 School of Sport, Exercise and Rehabilitation Sciences, College of Life and Environmental Sciences, 1724 University of Birmingham, Birmingham, UK

**Keywords:** Scapholunate instability, survey, rehabilitation, outcome measures, conservative treatment

## Abstract

**Introduction:**

Scapholunate instability is one of the most frequent types of wrist instability, but optimal management is not established. This research aims to identify current conservative management strategies for stage-one scapholunate instability and how these interventions are evaluated in the UK.

**Methods:**

A cross-sectional online survey of UK physiotherapists and occupational therapists with self-reported experience in the rehabilitation of stage-one scapholunate instability (ReSOS), was developed using the CROSS guideline and a clinical vignette. The frequency of treatment strategies was collated via a five-point Likert-type scale and evaluation strategies via fixed-response answers at three-to-six, seven-to-eleven and after 12 weeks post-injury. Data were analysed descriptively.

**Results:**

Forty-three electronic surveys were completed and analysed. Thirty physiotherapists and 13 occupational therapists responded, with 90% working in the NHS. Activity advice and education was the most frequently used treatment at all time-points (100%, 98%, 98%). Quick-DASH was most frequently used region-specific patient reported outcome measure at all time-points (72%, 60%, 67%).

**Discussion:**

Despite some identified themes, including neuromuscular rehabilitation strategies, the supporting evidence is limited in the ReSOS. It is unclear what rehabilitation and evaluation strategies are optimal and the development of a consensus on best practice is recommended.

## Introduction

Hand and wrist injuries not only constitute a substantial healthcare cost but also represent a considerable economic burden due to lost productivity.^1^ Societal costs are amplified as the peak incidence is in working age populations.^2,3^ Whilst the UK costs are unreported, $740 million (USD) was lost in healthcare and societal costs in the Netherlands in 2007.^4^ Scapholunate (SL) instability commonly occurs from a fall on an outstretched hand, resulting in injury or laxity of the SL ligaments and supporting structures.^5^ Symptoms can include localised radial dorsal wrist pain, clicking, reduced ability to weight bear and reduced grip.^6,7^

SL instability is one of the most frequent types of wrist instability.^8^ Incidence of SL tears is reported between 28% and 34% of wrists with cadaver investigation.^9^ However, although the in-vivo incidence of SL instability remains unknown,^10^ SL instability accounts for 20% of sprained wrists^11^ and in up to 64% of patients with concurrent distal radius fractures.^12^ Most injuries occur in men (68%) at a mean age of 39 years, at work (38%) or during sport (36%).^2^

SL instability is a spectrum, ranging from partial injury to the SL ligaments (stage-one) to complete disruption with cartilage loss (stage six).^13^ Common terms for stage-one SL instability includes pre-dynamic instability or partial ligament injury.^14^

Although surgical intervention is the mainstay of treatment for stages 2-6^15,16^ optimal management of stage-one SL instability has not been established.^17^ Surgical management of stage-one SL instability may include debridement, electrothermal shrinkage or dorsal capsulodesis.^13,18,19^ In recent years, the potential of neuromuscular rehabilitation in the conservative management of stage-one SL instability has been suggested as an appropriate treatment pathway.^20–22^ Clinical studies are, however, limited to case studies and service evaluations of stage-one SL instability only.^23–25^ Given the lack of current evidence base surrounding conservative management of stage-one SL instability, it is important to identify current practice.^26^ This is a crucial first step to determining a potential “best practice” treatment to evaluate in future research. There is no current published evidence supporting the conservative management of higher-grade SL injuries.

To the authors’ knowledge there are no previous studies investigating the rehabilitation and evaluation strategies used with stage-one SL instability. An online survey investigating the current rehabilitation and evaluation strategies in the conservative management of stage-one SL instability in UK clinical practice is warranted to investigate current practice and inform future practice and research.

## Aims

To determine contemporary conservative management and rehabilitation strategies of stage-one SL injuries in UK clinical practice.

### Primary research question

1. What are the current conservative management and rehabilitation strategies of stage-one SL instability in UK clinical practice?

### Secondary research question

2. How do clinicians measure effectiveness of current conservative management and rehabilitation strategies of stage-one SL instability in UK clinical practice?

## Methods

### Ethics

This study was approved by the Ethics Committee of School of Sport, Exercise and Rehabilitation Sciences at the University of Birmingham, United Kingdom (Reference: MCR2223_16).

### Questionnaire development and piloting

A cross-sectional online survey hosted by Microsoft Forms (Microsoft Office 365, University of Birmingham) was developed using a Consensus-Based Checklist for Reporting of Survey Studies (CROSS).^27^ Previously published musculoskeletal surveys^28–33^ influenced content including demographic data collected, use of Likert-type scales, use of a vignette, recruitment methods, and analytical techniques.

**Figure 1.**
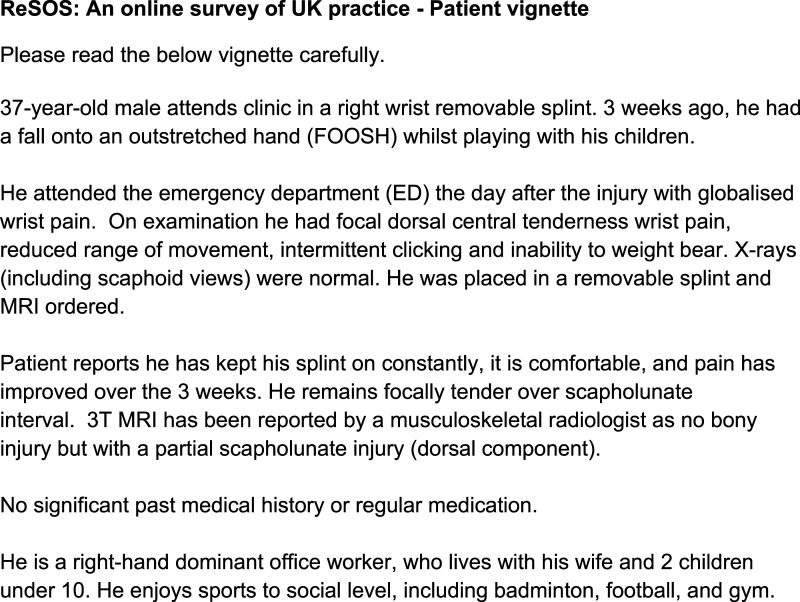
Clinical vignette.

The survey was designed with reference to a vignette representing a common patient presentation with stage-one SL instability. Clinical vignettes are valid tools for reflecting on clinical practice and clinical decision making.^34^ The vignette (Figure 1) was developed using known symptoms of stage-one SL instability^12^ (Table 1), existing case studies and vignettes.^23–25^ The vignette was reviewed by a multidisciplinary panel of consultant hand surgeons and post-graduate trained physiotherapists and occupational therapists, via a convenience sample at Sandwell and West Birmingham (SWBH) and University Hospitals Birmingham (UHB) NHS Foundation Trusts. Feedback included symptom clarification, specifying MRI magnetic field strength e.g. 1.5T or 3T, and reporting clinician and grammatical changes. This gained consensus on typical presentation and initial conservative management.

**Table 1.** Stages of SL instability.^13^

Stage	Injury description
1	Partial scapholunate ligament injury
2	Complete disruption with repairable ligament
3	Complete disruption with irreparable ligament but normal alignment
4	Complete disruption with irreparable ligament and reducible rotary subluxation of the scaphoid
5	Complete disruption with irreducible malalignment and intact cartilage
6	Complete disruption with cartilage loss

Literature searches were conducted on Medline, AMED, Embase and CINAHL (2010- 15/1/22) to establish current treatment and evaluation strategies within the literature using Boolean operators.^35^ Studies informed treatment and evaluation strategies included in the survey.

The survey was piloted via a convenience sample, consisting of post-graduate trained physiotherapists, occupational therapists from SWBH and UHB NHS Foundation Trusts and the research team. Changes suggested included reducing number of timeframes, reformatting questions and demographics collected. Feedback was acted upon and repiloted with the same sample, to agree the content.

To avoid having an incomplete data set, the survey could only be submitted after the respondent had completed all mandatory questions. The survey consisted of 27 questions in seven sections. Respondents were asked to confirm consent, study eligibility and that they would only submit one survey. As per clinical vignette (Figure 1) zero-to-three weeks timeframe reflected the pragmatic management of this injury from the emergency department to specialist clinic with MRI results in the NHS. The sections investigated the treatment strategies used in timeframes three-to-six weeks, seven-to-eleven weeks and 12 weeks onwards post injury in line with healing timeframes and common rehabilitation phases.^36–38^

A five-point qualitative Likert-type scale (Never, Rather infrequently, Some of the time, Quite often, Always)^39^ was used to determine frequency of use similar to previous studies.^31,32^ Likert-type scales are frequently used to measure attitudes or beliefs^40^ and have been widely used within surveys of practice within physiotherapy.^28,32,41^ Verbalised rating scales and scales with five-to-seven options have evidence of improved reliability and validity.^42^

Respondents were asked what evaluation strategies were used at three-to-six weeks, seven-to-eleven weeks and 12 weeks onwards post injury via fixed response answers. Listed items were established from previous literature search and included physical measures such as grip testing, patient reported outcome measures (PROMs) including Disabilities of the Arm, Shoulder and Hand^43^ (DASH), and patient reported experience measures (PREMs) such as patient satisfaction. A full copy of the survey is available in Supplementary Material (S1).

### Recruitment

The target population was Health and Care Professions Council registered physiotherapists and occupational therapists with self-reported experience in treating stage-one SL instability within the UK.

Exclusion criteria included non-registered therapists or clinicians and registered therapists without experience in the rehabilitation of SL instability and those who do not provide consent to participate.

The study was promoted via social media, emails to the professional networks and clinical interest groups including British Association of Hand Therapy (BAHT), interactive Chartered Society of Physiotherapy and Musculoskeletal Association of Chartered Physiotherapists. Reminders were sent weekly via social media and with 1 week remaining to email contacts. The study was open from 16^th^ January- 13th February 2023 (4 weeks) in line with previous studies.^31,33^ Whilst others have identified that there is minimal completion of e-surveys after this timeframe,^44^ this period also reflected the pragmatics of a student MSc project.

### Sample size

Due to a lack of published data on stage-one SL instability incidence in the UK, it was challenging to estimate population and sample size. Based on UK specialist interest group membership (BAHT=764), specialist nature of the condition and previous low recruitment rate in previous service evaluation,^24^ therapist population was estimated. From clinical experience, it was considered that not all BAHT members would have experience of treating SL instability and pragmatically that within regional hand therapy unit 25% of therapists treated SL instability: therefore it was estimated that there was a population of approximately 200 eligible therapists. Commonly reported response rates for online student surveys vary between below 10%–20%^45^ whilst others suggest a minimum of 12% response rate is acceptable for this population.^46^ Pragmatically a 25 % response rate (target 50 responses) was deemed reasonable.

### Analysis

Data was analysed descriptively using Microsoft Excel (Microsoft 365, license University of Birmingham). Likert-type items were analysed individually via percentage frequencies,^47–50^ in line with previous studies.^30–32^ The study aimed to identify interventions that are used in stage-one SL instability, therefore responses were grouped^32^ into “never & rather infrequently” to indicate those infrequently used and “quite often or always” to indicate those frequently used, similar to previous literature.^31,32^ Any free text responses to additional treatment and evaluation techniques were extracted from the survey and grouped, counted and reported in addition to original items with Likert scale (available in Supplementary Material).

## Results

### Response

A total of 43 surveys were completed and analysed.

### Sample characteristics

Seventy percent (30/43) of respondents were physiotherapists and 30% (13/43) were occupational therapists. Ninety percent (39/43) of respondents were employed in the NHS, and fifty-three percent (23/43) of respondents worked in a specialist upper limb or hand unit. Respondents were of a high Agenda for Change (AfC) banding and experienced, with 86% “AfC Band” seven or above (37/43): 65% (28/43) had over 10 years’ experience assessing and treating wrist and hand pathologies. Seventy percent (30/43) of respondents had formal post-graduate education. Sample characteristics are shown in Table 2.

**Table 2.** Respondent characteristics.

Respondent characteristics	Number of responses (%)
Profession	
Physiotherapist	30 (70)
Occupational therapist	13 (30)
Professional working area	
NHS - primary care	6 (14)
NHS - secondary care	8 (19)
NHS - specialist upper limb/Hands	23 (53)
NHS - orthopaedics	1 (2)
NHS - first contact practitioner	0 (0)
NHS - urgent care/Emergency dept	1 (2)
NHS - other	0 (0)
Clinical private practice	2 (5)
Clinical sport	2 (5)
Other	0 (0)
AfC Banding	
5	1 (2)
6	4 (9)
7	18 (42)
8a	18 (42)
8b	1 (0)
8c	0 (0)
9	1 (2)
Non-NHS banding	0 (0)
Clinical experience	
0-2	3 (7)
3-5	3 (7)
6-9	9 (21)
10+	28 (65)
Highest educational award	
Diploma (MCSP/MCOT)	1 (2)
BSc	12 (28)
Post graduate certificate	0 (0)
Post graduate diploma	5 (12)
MSc	24 (56)
PhD	1/43 (2)

### Frequency of conservative management strategies in stage-one SL instability

Respondents used a wide range of treatment strategies across all time-points; however, many were infrequently used. Results are presented in Table 3.

**Table 3.** Reported current conservative management strategies of stage-one SL instability in UK clinical practice.

	3-6 Weeks *N*, (%)			7-11 weeks *N*, (%)			12+ weeks *N*, (%)		
	Never/Rather infrequently	Some of the time	Quite often/Always	Never/Rather infrequently	Some of the time	Quite often/Always	Never/Rather infrequently	Some of the time	Quite often/Always
Immobilisation in POP	35 (81)	7 (16)	1 (2)	43 (100)	0 (0)	0 (0)	43 (100)	0 (0)	0 (0)
Immobilisation in orthotic (splint)	19 (44)	11 (26)	13 (30)	40 (93)	3 (7)	0 (0)	43 (100)	0 (0)	0 (0)
Orthotic and active ROM	7 (16)	9 (21)	27 (63)	28 (65)	8 (19)	7 (16)	39 (91)	2 (5)	2 (5)
Orthotic and DTM	17 (40)	9 (21)	17 (40)	24 (56)	7 (16)	12 (28)	32 (74)	5 (12)	6 (14)
AROM exercises	7 (16)	8 (19)	28 (65)	1 (2)	4 (9)	38 (88)	7 (16)	7 (16)	29 (67)
PROM exercises	33 (77)	7 (16)	3 (7)	10 (23)	15 (35)	18 (42)	14 (33)	10 (23)	19 (44)
DTM range of movement exercises	17 (40)	10 (23)	16 (37)	5 (12)	12 (28)	26 (60)	14 (33)	8 (19)	21 (49)
Oedema management	2 (5)	10 (23)	31 (72)	16 (37)	11 (26)	16 (37)	30 (70)	6 (14)	7 (16)
Activity advice & education	0 (0)	0 (0)	43 (100)	1 (2)	0 (0)	42 (98)	0 (0)	1 (2)	42 (98)
Joint sense position/kinaesthesia/Mirror therapy &/or laterality training	25 (58)	9 (21)	9 (21)	11 (26)	13 (30)	19 (44)	13 (30)	10 (23)	20 (47)
Global hand/wrist isotonic (concentric and/or eccentric) resistance training	30 (70)	5 (12)	8 (19)	6 (14)	14 (33)	23 (53)	1 (2)	5 (12)	37 (86)
Isometric training of APL, FCR, ECRL	14 (33)	7 (16)	22 (51)	6 (14)	8 (19)	29 (67)	1 (2)	5 (12)	37 (86)
Isokinetic training	33 (77)	9 (21)	1 (2)	25 (58)	6 (14)	12 (28)	19 (44)	4 (9)	20 (47)
Co-activation exercises	23 (53)	12 (28)	8 (19)	10 (23)	10 (23)	23 (53)	8 (19)	8 (19)	27 (63)
Whole body conditioning and/or kinetic chain optimisation	21 (49)	7 (16)	15 (35)	11 (26)	8 (19)	24 (56)	6 (14)	6 (14)	31 (72)
Perturbation training via progressive weight-bearing exercises	37 (86)	3 (7)	3 (7)	13 (30)	6 (14)	24 (56)	2 (5)	6 (14)	35 (81)
NWB perturbation training via gyroscope/rhythmic stabilisation/Slosh pipe	36 (84)	6 (14)	1 (2)	14 (33)	7 (16)	22 (51)	2 (5)	7 (16)	34 (79)
Plyometric training	40 (93)	2 (5)	1 (2)	20 (47)	13 (30)	10 (23)	7 (16)	7 (16)	29 (67)
Sport or work specific functional exercises	26 (60)	10 (23)	7 (16)	11 (26)	10 (23)	22 (51)	0 (0)	4 (9)	39 (91)

DTM, Dart Throwing Motion; AROM, Active range of movement; PROM, Passive range of movement; APL, Abductor pollicis longus; FCR, flexor carpi radialis, ECRL, extensor carpi radialis longus.

### Frequency of conservative management strategies in stage-one SL instability at three-to-six weeks post injury

Activity advice and education was used by all respondents (43/43, 100%). Frequently reported strategies included oedema management (31/43, 72%), active range of movement (AROM) exercises (28/43, 65%), use of an orthotic and AROM (27/43, 63%) and isometric training of abductor pollicis longus (APL), flexor carpi radialis (FCR), extensor carpi radialis longus (ECRL) (22/43, 51%).

Treatment strategies which respondents stated were used infrequently included plyometric exercises (40/43, 93%), perturbation training via progressive weightbearing exercises (37/43, 86%) or via gyroscope, rhythmic stabilisation or slosh pipe training (36/43, 84%), immobilisation in plaster of Paris (POP) (35/43%, 81%), passive range of movement (PROM) exercises (33/43, 77%) and isokinetic training (33/43, 77%).

Twenty-one percent (9/43) of respondents reported using other treatments than those compiled from the literature. Responses included taping (2/43, 5%), isometric brachioradialis and extensor capri ulnaris training (1/43, 2%), otherwise responses expanded on other listed strategies such as “proprioception”, “ROM” exercises or advice “don’t stop cardio exercise or strength training.”

### Frequency of conservative management strategies in stage-one SL instability at seven-to-eleven weeks post injury

The treatment strategy most reported was activity advice and education (42/43, 98%). Other modalities rated as being used frequently included AROM exercises (38/43, 88%), isometric training of APL, FCR, ECRL (29/43, 67%), Dart-throwing motion (DTM) ROM exercises (26/43, 60%) and global isotonic (concentric or eccentric) training (23/43, 53%).

Strategies rated as being used infrequently included immobilisation in POP (43/43, 100%), immobilisation in orthotic, (40/43, 93%), orthotic and AROM (28/43, 65%) and orthotic and DTM (24/43, 56%). Despite respondents using more resistance training, isokinetic training (25/43, 58%) and plyometric training (20/43, 47%) remained infrequently used.

Free-text responses included “FCR” or “weightbearing” exercises, “Pilates” or “movement patterning.”

### Frequency of conservative management strategies in stage-one SL instability at 12+ weeks post injury

Activity advice and education (42/43, 98%) remained the most used strategy. Other treatment strategies frequently reported included sport or work specific functional exercises (39/43, 91%), global hand and wrist isotonic resistance training (37/43, 86%), perturbation training via progressive weightbearing exercises (35/43, 81%), non-weightbearing (NWB) perturbation training via such as gyroscopes/rhythmic stabilisation or slosh pipes (34/43, 79%) and whole body/kinetic chain optimisation (31/43, 72%).

Free-text responses included “corticosteroid injection” or self-management strategies including “pain diary.”

### Frequency of treatment evaluation strategies in stage-one SL instability

**Figure 2.**
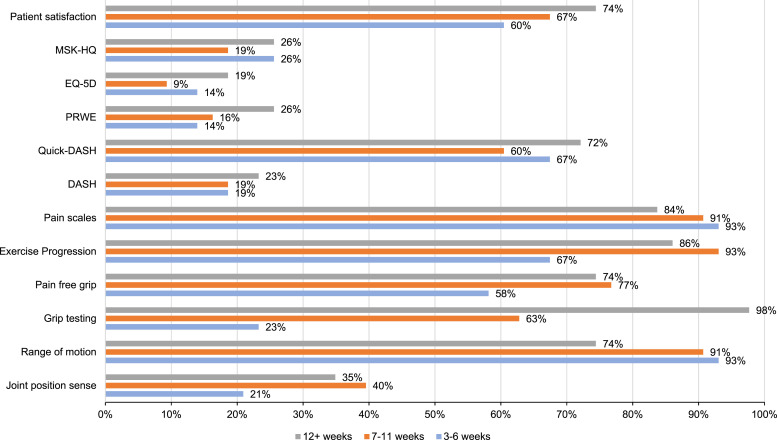
Percentage frequency of used evaluation strategies at 3-6, 7-11 and 12+ weeks (DASH = Disabilities of the Arm, Shoulder and Hand, QuickDASH = Quick Disabilities of the Arm, Shoulder and Hand, PRWE = Patient-Rated Wrist Evaluation, EQ-5D = EuroQol-5D, Musculoskeletal Health Questionnaire).

Several evaluation strategies including physical testing, PROMs and PREMs were used to evaluate treatment effectiveness, although some were reported infrequently. Results are shown in Figure 2. Quick-DASH^51^ was the most used region-specific PROM across all time-points (29/43, 67% vs. 26/43, 60% vs. 31/43, 72%).

### Frequency of treatment evaluation strategies in stage-one SL instability at three-to-six weeks post injury

Range of motion (ROM) was the most used clinician-reported outcome assessment (40/43, 93%), followed by exercise progression (25/43, 67%) and pain-free grip (25/43, 58%). Pain scales were reported as frequently used (40/43, 93%) as were patient satisfaction scales (26/43, 60%).

### Frequency of treatment evaluation strategies in stage-one SL instability at seven-to-eleven weeks post injury

Exercise progression (40/43, 93%), ROM (39/43, 91%), pain scales (39/43, 91%) and pain free grip (33/43, 77%) were again the most used evaluation strategies and patient satisfaction remained regularly used (29/43, 69%).

### Frequency of treatment evaluation strategies in stage-one SL instability at 12+ weeks post injury

Maximum grip testing (42/43, 98%) was the most used evaluation strategy. Exercise progression (37/43, 86%), ROM (32/43, 74%), pain-scales (36/43, 84%) and pain-free grip (32/43, 74%) were again regularly used evaluation strategies. The reported use of patient satisfaction remained similar (32/43, 74%).

### Other suggested treatment evaluation strategies in stage-one SL instability

Respondents (16/43, 37%) reported various other evaluation strategies, whilst most (12/16, 75%) of these respondents did not report a specific time frame for implementation. Reported evaluation strategies included PROMs such as “Patient Specific Function Scale”^52^ (PSFS), physical measures such as “weightbearing tolerance… via Jamar or scales” or functional goals such as “return to work” or “return to sport.”

## Discussion

**Figure 3.**
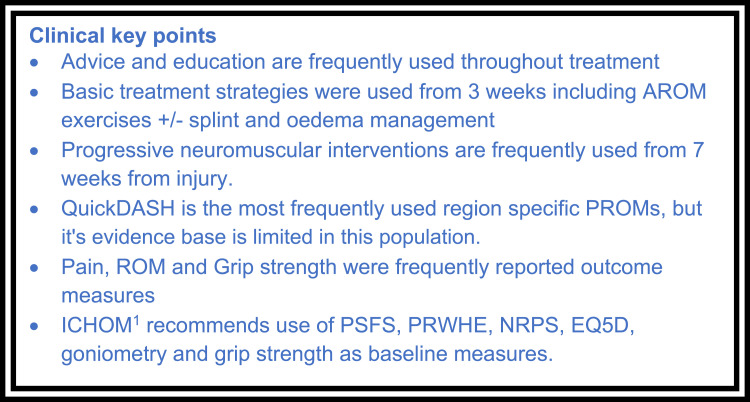
Clinical key points. PSFS, Patient specific functional scale; NPRS, numerical pain rating scale; EQ5D, EuroQol-5D, Musculoskeletal Health Questionnaire; PRWHE, patient-rated wrist and hand evaluation; QuickDASH, Quick Disabilities of the Arm, Shoulder and Hand; AROM, active range of movement exercises; ROM, Range of movement.

This online survey has reported the rehabilitation and evaluation strategies of stage-one SL injuries in UK clinical practice of physiotherapists and occupational therapists. Clinical key points are summarised in Figure 3.

### Analysis of reported conservative management strategies in stage-one SL instability

Activity advice and education was the most frequently used treatment strategy across all time-points. Patient advice and education provides a means for therapists to deliver information.^53^ Costa et al.,^54^ in a longitudinal cohort study evaluating a multimodal digital rehabilitation programme showed over a 50% improvement in pain and QuickDASH in 187 patients with hand and wrist pain after an evidence based education programme. In wider therapy practice, advice and education strategies are accepted as an essential part of therapy practice,^55,56^ and integral to effective patient care.^57^ Education strategies have been shown to improve exercise adherence,^58^ enhance patient self-efficacy,^59^ self-management skills^60^ and provide positive outcomes in relation to pain, disability and function.^61^ However, more work is needed to evaluate specific educational to strategies to SL instability patients.

DTM has previously been advocated as safe in early rehabilitation in SL injuries,^62,63^ as it has been argued that there is minimal movement of the SL joint.^64–66^ The practical application of DTM in the conservative management of stage-one SL instability is limited to use in small case-based studies^23,25,67^ but demonstrated positive outcomes in patient reported disability. DTM AROM exercises were more frequently reported at seven-to-eleven weeks post injury, suggesting clinicians were potentially progressing rehabilitation to align with soft tissue healing timeframes.^36,37^ Despite DTM exercises being frequently used at weeks seven-to-eleven there has been some recent research to indicate caution with its use. One scoping review,^9^ identified four cadaveric and one in- vivo study investigating the biomechanics of DTM concluded that end-range radial extension and ulnar flexion may induce movements and forces that dissociate the injured SLIL and a orthotic may be able to limit movement to mid-range and that more research is required. The use of an orthotic with DTM was infrequently reported by respondents across all time-points; and perhaps due the low quality evidence supporting its application does not seem to have transitioned into UK practice during this time point. The use of DTM exercises in the rehabilitation of stage-one SL instability therefore remains unclear and further research is needed to understand it’s role and effect in this population.

Interventions between three-to-six weeks included “basic”^20^ rehabilitation strategies suggested for acute injury^68^ and within a suggested framework for this injury^69^ such as oedema management and AROM. There was commonality in the use of progressive conscious neuromuscular strategies, such as resistance and conditioning exercises and unconscious neuromuscular strategies,^20,70^ such as perturbation training, at 7 weeks onwards post injury aligning with soft tissue healing timeframes.^36,37^

Isometric training of APL, FCR and ECRL has been recommended as a rehabilitation strategy for SL instability^21^ and was more frequently reported at 7 weeks onwards post injury. Cadaveric studies^71^ (*n* = 10) have shown that isolated loading of APL, FCR or ECRL, supinates the scaphoid reducing the SL diastasis, and thus improving the carpal alignment. However this reduction has not been imaged with in-vivo subjects and must therefore be interpreted with caution. Experimental studies have suggested these muscles are also activated as part of early monosynaptic “protective reflexes” when the dorsal SL ligament is stressed^72^ and supports their use in rehabilitation.

The reported frequency of the use of complex exercise strategies, resistance training and functional interventions, increased at 12 weeks onwards compared to earlier time-points. This included sport or work specific functional exercises, global hand and wrist isotonic resistance training, perturbation training via progressive weightbearing exercises, or NWB perturbation training via gyroscopes/rhythmic stabilisation or slosh pipes and whole body/kinetic chain optimisation. Theoretical application of progressive neuromuscular rehabilitation programmes for SL instability have been suggested in the literature^20,70^ and are common components in the progressive rehabilitation of other upper limb instabilities^73–75^ and return to sport.^76^ Clinical application of such training strategies in SL instability is limited to case work^23,25^ and a small service evaluation.^24^ Positive results in patient reported disability, pain and function were shown in all studies. However, the studies total only 13 wrists which limits their findings and external validity to clinical practice.

This study demonstrates that progressive neuromuscular interventions are frequently reported by a sample of 43 UK physiotherapists and occupational therapists in the treatment of stage-one SL instability at 7 weeks onwards post injury as suggested by theoretical frameworks.^20^ However, the evidence underpinning suggested strategies for stage-one SL instability is based on low quality evidence and should be interpreted with caution.

The survey’s results can inform treatment and the design of much needed research in this patient group. Future research should consider the development of consensus based, best practice treatment guidelines using methods such as a Delphi. This could inform the intervention arm of a future randomised clinical trial to evaluate therapy in this population.

### Analysis of reported treatment evaluation strategies in stage one SL instability

Measuring the outcomes of clinical care to ensure optimal patient outcomes, cost effectively, is a key aspect of value-based, personalised healthcare.^77,78^ This survey is the first study to investigate the current evaluation strategies used in stage-one SL instability.

A standard set for outcome measurement in hand and wrist conditions was recently developed via international consensus of hand surgeons, therapists, and researchers using International Consortium for Health Outcomes Measurement (ICHOM) standardised methods.^79^ The “extended” wrist track in ICHOM includes wrist instability and the recommended outcomes specific to wrist pathology at baseline, 3 months and 12 months.

Traditionally treatment evaluation, within therapy, has focused on physical testing such as grip or AROM to evaluate patient outcome.^80^ ICHOM^79^ recommended clinician-reported outcome measures included goniometry to measure ROM and grip strength. Both were frequently reported, alongside exercise progression across all time points in our study. Maximum grip testing was used more at 7 weeks onwards post injury and may reflect the increased use of resistance training at these time-points and soft tissue healing timeframes.^36,37^ Outcomes used within the limited clinical studies^23–25^ in the SL population are heterogeneous in nature but all used grip measurements. However, it is unclear which, if any physical measures are optimal and no studies validating their use in this population exist.

Clinician-reported outcome measures may not consider function and psychosocial outcomes important to patients^78^ Musculoskeletal services in the UK are now expected to use validated PROMs and PREMs to assess musculoskeletal health, review progress, feedback on care and assist in benchmarking services.^81,82^ ICHOM^79^ recommendations included the use of PSFS, pain scales, PRWHE and pateint satisfaction. Our own study reported frequent use of pain scales, patient satisfaction, whilst PSFS was mentioned in free text responses.

The Patient-Rated Wrist Evaluation (PRWE)^83^ is the key region-specific PROM recommended by ICHOM. It was also included in a recent overview of systematic reviews (81) evaluating psychometric properties of PROMs for hand and wrist disorders and demonstrated good to excellent reliability but weak to moderate validity.^80,84,85^ Despite the recommended use of PRWHE,/PRWE, it was not frequently reported at all time points and may reflect it’s specialist nature and potential availability, especially in UK NHS practice.

ICHOM^79^ does not recommend the use of the QuickDASH, in contrast to the findings in this UK based survey. Respondents frequently used the Quick-DASH across all time-points to evaluate patient treatment. The psychometric properties of the Quick-DASH in evaluating SL instabilities remains unknown. However, in contrast to a specialist measure such as PRWHE/PRWE, the Quick-DASH, is easily available for NHS clinicians and can be used for multiple upper limb conditions. With most practice occurring in a resource limited NHS, a pragmatic approach seems to be superseding ICHOM recommendations^79^ or may reflect therapists lack of awareness of the core outcome sets.

Content validity is especially important in PROMs since how potential respondents interact with items depends on a variety of factors related to the PROMs content, and comprehensiveness.^86^ Wormald et al., 2019,^87^ a review of systematic reviews, argues that whilst the Quick-Dash has some of the most published psychometric data, it lacks content validity and some psychometric properties are not adequately described in the published literature. Of the eight included studies evaluating the Quick-DASH only three studies included wrist trauma patients.^88–90^ Of these studies, one^88^ did not use the standard 11 item Quick-DASH,^51^ and another study^89^ included only thirteen hand “trauma” patients but did not report specific pathologies included. The third study^90^ (*n* = 35) included only two distal radius fractures and two wrist “sprains”, with majority of pathologies being “rotator cuff tears” limiting their findings to a wrist trauma or SL instability populations.

Despite respondents reporting use of Quick-DASH, it is unclear if Quick-DASH is the most appropriate PROM in this population and cognitive interviewing of patients with SL instability to establish content validity is suggested.^86^

The uncertainty surrounding optimal evaluation strategies is not unique to SL instability, but indicative of musculoskeletal practice in the UK, with no established standardisation of outcomes or benchmarking strategies.^81^ Future research of evaluation strategies should be informed by this survey’s results of current practice and the standardised sets in the literature, to produce standardised outcomes that are feasible in practice, can improve patient care and allow meaningful benchmarking.^81^

### Limitations

The survey reflected a mix of UK physiotherapists and occupational therapists working in multiple clinical settings, thus increasing the likelihood that the findings of this study will be transferable. However, it was challenging to estimate the sample size required due to unknown incidence of the condition and therapist population treating stage-one SL instabilities. There were 43 survey respondents in the cross-sectional survey. This low sample size is likely to under-represent the number of UK physiotherapists and occupational therapists who manage SL instability in clinical practice.

This is a survey of UK practice; however, authors were unable to electronically limit respondents to the UK, or limit respondents to completing the survey once, potentially biasing results. However, respondents were informed of the study’s criteria and confirmed eligibility and that they would only submit one response as part of the consent process. Furthermore, most respondents were physiotherapists working in the NHS and may not reflect the practice of non-NHS clinicians.

## Conclusion

This is the first study to investigate the current conservative management and evaluation strategies used in stage-one SL instability in UK clinical practice. The survey presents frequently reported treatments including advice and education and progressive neuromuscular treatment strategies, which is comparable with the limited clinical studies and theoretical frameworks in the literature.

The Quick-DASH was the most frequently used PROMs by respondents, and several physical measures including, grip and ROM are used to evaluate patient care. However, the research supporting their use is limited. Future research should develop consensus-based guidelines on best practice using methods such as a Delphi. Current hand and wrist core outcome sets need to be evaluated to further assess psychometric properties of the outcome measurement instruments for stage-one SL instability.

## Supplemental Material

^91–98^Supplemental Material - Rehabilitation of stage-one scapholunate instability (ReSOS): An online survey of UK practice. *R*e*SOS:* A survey of UK practice Supplemental Material for Rehabilitation of stage-one scapholunate instability (ReSOS): An online survey of UK practice. *R*e*SOS:* A survey of UK practice by Martin K Holmes, Caroline Miller and Michael Mansfield in Hand Therapy.
