# Supplementary material for: Rehabilitation of stage-one scapholunate instability (ReSOS): An online survey of UK practice
Source: Hand Ther. 2024 Aug 16;29(4):175–87. doi: 10.1177/17589983241268056 (PMC11500232; doi:10.1177/17589983241268056)
Supplement: 91–98Supplemental Material - Rehabilitation of stage-one scapholunate instability (ReSOS): An online survey of UK practice. ReSOS: A survey of UK practice [file sj-pdf-1-hth-10.1177_17589983241268056.pdf]

Supplementary File A - Consensus-Based Checklist for Reporting of Survey Studies (CROSS)[28]

Section/topic	Item	Item description	Reported on page #
**Title and abstract**			
Title and abstract	1a	State the word "survey" along with a commonly used term in title or abstract to introduce the study's design.	1, 3-4
	1b	Provide an informative summary in the abstract, covering background, objectives, methods, findings/results, interpretation/discussion, and conclusions.	3-4
**Introduction**			
Background	2	Provide a background about the rationale of study, what has been previously done, and why this survey is needed.	5-6
Purpose/aim	3	Identify specific purposes, aims, goals, or objectives of the study.	7
**Methods**			
Study design	4	Specify the study design in the methods section with a commonly used term (e.g., cross-sectional or longitudinal).	8
Data collection methods	5a	Describe the questionnaire (e.g., number of sections, number of questions, number and names of instruments used).	9-10
	5b	Describe all questionnaire instruments that were used in the survey to measure particular concepts. Report target population, reported validity and reliability information, scoring/classification procedure, and reference links (if any).	8-11

	5c	Provide information on pretesting of the questionnaire, if performed (in the article or in an online supplement). Report the method of pretesting, number of times questionnaire was pre-tested, number and demographics of participants used for pretesting, and the level of similarity of demographics between pre-testing participants and sample population.	9-10
	5d	Questionnaire if possible, should be fully provided (in the article, or as appendices or as an online supplement).	Supplementary File C
Sample characteristics	6a	Describe the study population (i.e., background, locations, eligibility criteria for participant inclusion in survey, exclusion criteria).	10
	6b	Describe the sampling techniques used (e.g., single stage or multistage sampling, simple random sampling, stratified sampling, cluster sampling, convenience sampling). Specify the locations of sample participants whenever clustered sampling was applied.	10
	6c	Provide information on sample size, along with details of sample size calculation.	11
	6d	Describe how representative the sample is of the study population (or target population if possible), particularly for population-based surveys.	11
Survey administration	7a	Provide information on modes of questionnaire administration, including the type and number of contacts, the location where the survey was conducted (e.g., outpatient room or by use of online tools, such as SurveyMonkey).	10
	7b	Provide information of survey's time frame, such as periods of recruitment, exposure, and follow-up days.	10-11

	7c	Provide information on the entry process:<br>–>For non-web-based surveys, provide approaches to minimize human error in data entry.<br>–>For web-based surveys, provide approaches to prevent “multiple participation” of participants.	9
Study preparation	8	Describe any preparation process before conducting the survey (e.g., interviewers’ training process, advertising the survey).	10-11
Ethical considerations	9a	Provide information on ethical approval for the survey if obtained, including informed consent, institutional review board [IRB] approval, Helsinki declaration, and good clinical practice [GCP] declaration (as appropriate).	8
	9b	Provide information about survey anonymity and confidentiality and describe what mechanisms were used to protect unauthorized access.	9-10
Statistical analysis	10a	Describe statistical methods and analytical approach. Report the statistical software that was used for data analysis.	11
	10b	Report any modification of variables used in the analysis, along with reference (if available).	11
	10c	Report details about how missing data was handled. Include rate of missing items, missing data mechanism (i.e., missing completely at random [MCAR], missing at random [MAR] or missing not at random [MNAR]) and methods used to deal with missing data (e.g., multiple imputation).	11<br>9
	10d	State how non-response error was addressed.	NA
	10e	For longitudinal surveys, state how loss to follow-up was addressed.	NA
	10f	Indicate whether any methods such as weighting of	NA

		items or propensity scores have been used to adjust for non-representativeness of the sample.	
	10g	Describe any sensitivity analysis conducted.	NA
**Results**			
Respondent characteristics	11a	Report numbers of individuals at each stage of the study. Consider using a flow diagram, if possible.	12
	11b	Provide reasons for non-participation at each stage, if possible.	NA
	11c	Report response rate, present the definition of response rate or the formula used to calculate response rate.	NA
	11d	Provide information to define how unique visitors are determined. Report number of unique visitors along with relevant proportions (e.g., view proportion, participation proportion, completion proportion).	NA
Descriptive results	12	Provide characteristics of study participants, as well as information on potential confounders and assessed outcomes.	12 & Table 2
Main findings	13a	Give unadjusted estimates and, if applicable, confounder-adjusted estimates along with 95% confidence intervals and p-values.	NA
	13b	For multivariable analysis, provide information on the model building process, model fit statistics, and model assumptions (as appropriate).	NA
	13c	Provide details about any sensitivity analysis performed. If there are considerable amount of missing data, report sensitivity analyses comparing the results of complete cases with that of the imputed dataset (if possible).	NA
**Discussion**			

Limitations	14	Discuss the limitations of the study, considering sources of potential biases and imprecisions, such as non-representativeness of sample, study design, important uncontrolled confounders.	21-22
Interpretations	15	Give a cautious overall interpretation of results, based on potential biases and imprecisions and suggest areas for future research.	16-22
Generalizability	16	Discuss the external validity of the results.	16-22
**Other sections**			
Role of funding source	17	State whether any funding organization has had any roles in the survey's design, implementation, and analysis.	1
Conflict of interest	18	Declare any potential conflict of interest.	1
Acknowledgements	19	Provide names of organizations/persons that are acknowledged along with their contribution to the research.	1

Supplementary file B - Glossary: *Re*habilitation of *Stage-one S*capholunate instability (ReSOS): An online survey of UK practice

Term	Description
Coactivation exercises	Exercises facilitating the simultaneous contraction of agonist and antagonist muscles across a joint.[21]
Concentric muscle contraction / training	Exercises designed to strengthen the muscle while it is shortening, usually because of an opposing load.[91]
Dart throwing motion (DTM) exercises	Range of movement exercises across the plane in which wrist functional oblique motion occurs, specifically from radial extension to ulnar flexion.[92]
Eccentric muscle contraction / training	Exercises designed to strengthen the muscle while it is lengthened, usually because of an opposing load.[91]

Graded Motor Imagery; Mirror therapy and laterality training	The first stage of graded motor imagery includes laterality reconstruction, in which patients view various images of body parts to determine whether the image portrays the right or left side.<br>The second stage targets activation of the primary motor cortex, requiring the patient to imagine moving the involved body part. This has been shown to activate the same areas of the cortex associated with movement of the body part.<br>The final stage involves having the patient watch the unaffected body part moving in a mirror to "trick" the brain into thinking the affected body part is moving in a pain-free manner.[93] The patient sits in front of a mirror that is oriented parallel to his midline blocking the view of the (affected) limb, positioned behind the mirror. When looking into the mirror, the patient sees the reflection of the unaffected limb positioned as the affected limb. This arrangement is suited to create a visual illusion whereby movement of or touch to the intact limb may be perceived as affecting the paretic or painful limb.[94]
Gyroscope training	A gyroscope exerciser, such as NSD Powerball, (RPM Sports, Tipperary, Ireland), is a hollow sphere that contains in the interior a rotor of 200 grammes of weight with an eccentric mass located two centimetres away from its axis. This internal cylinder rotates around an axis which is perpendicular to the main axis. The internal rotor moves not so much because of its fixed weight (single weight 280 grammes) but by the generated centrifugal force. When the internal rotor is accelerated, generates a torsion force that causes a turn in the perpendicular plane, and because the eccentric disposition of its mass, a rotational force to the rotor is generated up to10,000 revolutions per minute. The gyroscope accelerates by means of movements of wrist rotation. As the speed of the rotor of the gyroscope increases, the centrifugal force increases and, therefore, the necessity of muscular control becomes increasingly bigger.[95]
Isokinetic training	A muscle contraction performed at constant angular speed and is thus independent of degree of muscle amplitude and velocity. It allows for a controlled training, enhances muscle strength throughout joint motion, and promotes endurance, using a commercial isokinetic exerciser. [21]
Isometric resistance training	Isometric exercise is when an active muscle contraction is performed at a fixed joint angle.[91] 1993).
Joint sense position	The ability to accurately reproduce a specific joint angle.[21]
Kinaesthesia	The ability to sense motion of a joint (or a limb).[21]
Kinetic chain	The sequencing of required physiologic muscle activations in the upper and lower extremity to result in an integrated biomechanical task is known as the kinetic chain. Training to improve the proximal to distal function required for tasks is considered kinetic chain optimisation or

	rehabilitation.[96] For example, shoulder exercises for throwers in with wrist pain.
Perturbation training	Balance or Reactive muscle activation exercises aimed to restore normal neuromuscular reflex patterns that exist in a normal joint[21] and includes controlled unpredictable forces directed to an unstable surface or mechanism for improved efficiency of stabilising muscular contractions. [97]
Plyometric training	Plyometric exercise is identified using the stretch shortening cycle. This involves the stretch of the muscle-tendon unit immediately followed by shortening. [98]
Stages of Scapholunate Instability	Stage 1: Partial scapholunate ligament injury<br>Stage 2: Complete disruption with repairable ligament<br>Stage 3: Complete disruption with irreparable ligament but normal alignment<br>Stage 4: Complete disruption with irreparable ligament and reducible rotary subluxation of the scaphoid<br>Stage 5: Complete disruption with irreducible malalignment and intact cartilage<br>Stage 6: Chronic SLIL disruption with cartilage loss. [14]

Supplementary file C – Copy of MS Form - *R*ehabilitation of *Stage-one S*capholunate instability: An online survey of UK practice.

***R*ehabilitation of *Stage-one S*capholunate instability (ReSOS): An online survey of UK practice**

What is this study about?

Thank you for taking the time to consider this survey about the treatment and rehabilitation strategies used by physiotherapist and occupational therapists when treating stage 1 Scapholunate instability. Stages of Scapholunate instability are described in the glossary. Identifying the treatment and rehabilitation strategies used within this condition will inform the current limited evidence base on this condition and help shape future research agendas. It should take you approximately 15 minutes to complete.

You have been invited to participate in this study because you have accessed it via social media, your professional networks, or UK based specialist clinical interest group.

Please read the Participant Information Sheet (link below) and ask any questions about anything you do not understand or want to know more about. Participation in this research is voluntary and you can withdraw without consequence prior to submitting your answers, by exiting the survey.

The Participation Information Sheet is available at the following link:

https://acrobat.adobe.com/link/track?uri=urn:aaid:scds:US:67a1ee38-295a-3ee3-b7b1-07fcbf6e35dc

The glossary is available at the following link:

https://acrobat.adobe.com/link/track?uri=urn:aaid:scds:US:de8116eb-e4ea-31af-99b2-b79487e0c03b

Section 1: ReSOS: An online survey of UK practice - Online consent form

Online consent form -
MSc Advanced Manipulative Physiotherapy School of Sport, Exercise and Rehabilitation Sciences, University of Birmingham, UK

Study Title: *R*ehabilitation of *Stage-one S*capholunate instability (ReSOS): An online survey of UK practice

Primary Investigator - Mr. Martin Holmes (mkh388@student.bham.ac.uk)
Study Supervisor - Mr. Michael Mansfield (m.mansfield@bham.ac.uk)

This study was reviewed by the Ethics Committee of School of Sport, Exercise

and Rehabilitation Sciences at the University of Birmingham. (Approved ethics number: MCR2223_16.)

By giving your consent to participate in this study you are confirming you:

- Understand what you have read

- Agree to take part in the research study as outlined below

- Agree to use of your personal information as described below

- You have read and downloaded a copy of the Participation Information Sheet to keep.

The Participation Information Sheet is available at the following link:

https://acrobat.adobe.com/link/track?uri=urn:aaid:scds:US:67a1ee38-295a-3ee3-b7b1-07fcbf6e35dc

This study was reviewed by the Ethics Committee of School of Sport, Exercise and Rehabilitation Sciences at the University of Birmingham. (Approved ethics number: MCR2223_16.)

1. I confirm that I have read and understood the participant information sheet for this study. I have had opportunity to consider the information and received satisfactory answers to any questions which I have had.

   ☐ Yes
   ☐ No and exit study.

2. I confirm I have voluntarily agreed to participate in the study. I understand I can refuse to take part and can withdraw from the study at any time up to form submission, without having to give reason and without penalty, by exiting the survey.

   ☐ Yes
   ☐ No and exit study.

3. I consent to the processing of my personal information for the purposes explained in the participant information sheet.

   ☐ Yes
   ☐ No and exit study.

4. I understand that this survey is not a test of knowledge and there are no right or wrong answers. I understand that although direct quotations may be used

in publications, confidentiality and anonymity will be maintained and it will not be possible to identify me in any research outputs.

- ☐ Yes
- ☐ No and exit study.

5. I understand that this survey is not a test of knowledge, there are no right, or wrong answers and this survey is simply trying to establish current practice.

   - ☐ Yes
   - ☐ No and exit study.

6. I understand that once submitted I will be unable to withdraw, as the primary investigator will be unable to retrieve my anonymised data.

   - ☐ Yes
   - ☐ No and exit study.

7. Are you a HCPC registered physiotherapist or occupational therapist practicing in the UK with experience of treating or rehabilitating stage-one Scapholunate instability?

   - ☐ Yes – Physiotherapist
   - ☐ Yes – Occupational Therapist
   - ☐ No and exit study.

8. After considering all of the above, I agree to take part in the above study and will
   submit **one** completed survey only.

   - ☐ Yes
   - ☐ No and exit study.

Section 2 - ReSOS: An online survey of UK practice – Demographics

9. Please indicate your professional working area. If you work across multiple areas, please select the working area where you spend most time treating scapholunate instability.

- ☐ NHS – Primary Care
- ☐ NHS – Secondary Care
- ☐ NHS – Specialist Upper Limb / Hand Unit
- ☐ NHS – Orthopaedics

- ▢ NHS – First Contact Practitioner
- ▢ NHS – Urgent Care / Emergency Department
- ▢ NHS – Other
- ▢ Clinical Private Practice
- ▢ Clinical Sport
- ▢ Other

10. Please state the other sector you work in. If you work across multiple areas, please select the working area where you spend most time treating scapholunate instability.

    ____

11. Please indicate your current Agenda for Change (AfC) NHS banding, if applicable. If you have multiple bandings, please select the band where you spend most of your time.

- ▢ AfC Band 5
- ▢ AfC Band 6
- ▢ AfC Band 7
- ▢ AfC Band 8a
- ▢ AfC Band 8b
- ▢ AfC Band 8c
- ▢ AfC Band 9
- ▢ Non-NHS clinician

12. How many years have you worked (as a registered Occupational Therapist/ Physiotherapist) in a clinical setting assessing and treating people with wrist and hand pathologies?

- ▢ 0-2
- ▢ 3-5
- ▢ 6-10
- ▢ 10+

13. How much of your current clinical working time do you spend assessing and treating wrist and hand pathology?

- ▢ Less than 10%
- ▢ 11-24%
- ▢ 25-49%
- ▢ 50-74%

- ▢ 75-99%
- ▢ 100%

14. What is your highest educational award?

- ▢ Diploma (MCSP / MCOT)
- ▢ BSc / Equivalent
- ▢ Post Graduate Certificate
- ▢ Post Graduate Diploma
- ▢ MSc / Equivalent
- ▢ PhD / Equivalent

Section 3 - ReSOS: An online survey of UK practice - Patient vignette

Please read the below vignette carefully.

37-year-old male attends clinic in a right wrist removable splint. 3 weeks ago, he had a fall onto an outstretched hand (FOOSH) whilst playing with his children.

He attended the emergency department (ED) the day after the injury with globalised wrist pain. On examination he had focal dorsal central tenderness wrist pain, reduced range of movement, intermittent clicking and inability to weight bear. X-rays (including scaphoid views) were normal. He was placed in a removable splint and MRI ordered.

Patient reports he has kept his splint on constantly, it is comfortable, and pain has improved over the 3 weeks. He remains focally tender over scapholunate interval. 3T MRI has been reported by a musculoskeletal radiologist as no bony injury but with a partial scapholunate injury (dorsal component).

No significant past medical history or regular medication.

He is a right-hand dominant office worker, who lives with his wife and 2 children under 10. He enjoys sports to social level, including badminton, football, and gym.

Using the information available in the vignette as a reference, please answer the following questions.

ection 4 - Rehabilitation during 3 to 6 weeks post injury

Using the information available in the vignette as a reference, **please consider treatment strategies you would use for this patient in weeks 3 till 6 weeks post injury.**

A copy of the vignette is available to download, if required, on the below link.

https://acrobat.adobe.com/link/track?uri=urn:aaid:scds:US:176cd50c-9972-3cdd-9de5-02375d3cfbda

Glossary of terms, if required, is available at the link below:

https://acrobat.adobe.com/link/track?uri=urn:aaid:scds:US:de8116eb-e4ea-31af-99b2-b79487e0c03b

15. Rehabilitation during 3 to 6 weeks post injury: What rehabilitation or management strategies would you recommend for this patient in **weeks 3 to 6 post injury?**

	Never	Rather infrequently	Some of the time	Quite often	Always
Immobilisation in POP	☐	☐	☐	☐	☐
Immobilisation in Orthotic (splint)	☐	☐	☐	☐	☐
Orthotic and active ROM	☐	☐	☐	☐	☐
Orthotic and Dart Throwing Motion (DTM)	☐	☐	☐	☐	☐
Active range of movement (AROM) exercises	☐	☐	☐	☐	☐
Passive range of movement (PROM) exercises	☐	☐	☐	☐	☐
DTM range of movement exercises	☐	☐	☐	☐	☐
Oedema management	☐	☐	☐	☐	☐
Activity advice & education	☐	☐	☐	☐	☐
Joint sense position / kinaesthesia / Mirror therapy and/or laterality training	☐	☐	☐	☐	☐

Global hand/wrist isotonic (concentric and /or eccentric) resistance training	▢	▢	▢	▢	▢
Isometric training of abductor pollicis longus (APL), flexor carpi radialis (FCR), extensor carpi radialis longus (ECRL)	▢	▢	▢	▢	▢
Isokinetic training	▢	▢	▢	▢	▢
Co-activation exercises	▢	▢	▢	▢	▢
Whole body conditioning and/or Kinetic chain optimisation	▢	▢	▢	▢	▢
Perturbation training via progressive weight-bearing exercises	▢	▢	▢	▢	▢
Perturbatio*n* training via Gyroscope and/or rhythmic stabilisation and/or Slosh pipe training	▢	▢	▢	▢	▢
Plyometric training	▢	▢	▢	▢	▢
Sport or work specific functional exercises	▢	▢	▢	▢	▢

16. Are there any other treatment strategies (that are not listed above) that you would implement with this patient vignette **in weeks 3 to 6 post injury?**

- ▢ Yes
- ▢ No

17. What are the other treatment strategies (that are not listed above) that you would implement with this patient vignette **in weeks 3 to 6 post injury?**

____

Section 5 - Rehabilitation during 7 to 11 weeks post injury

Using the information available in the vignette as a reference, **please consider treatment strategies you would use for this patient during 7 to 11 weeks post injury.**

A copy of the vignette is available to download, if required, on the below link.

https://acrobat.adobe.com/link/track?uri=urn:aaid:scds:US:176cd50c-9972-3cdd-9de5-02375d3cfbda

Glossary of terms, if required, is available at the link below:

https://acrobat.adobe.com/link/track?uri=urn:aaid:scds:US:de8116eb-e4ea-31af-99b2-b79487e0c03b

18. **Rehabilitation during 7 to 11 weeks post injury:** What rehabilitation or management strategies would you recommend for this patient **during 7 to 11 weeks post injury?**

	Never	Rather infrequently	Some of the time	Quite often	Always
Immobilisation in POP	▢	▢	▢	▢	▢
Immobilisation in Orthotic (splint)	▢	▢	▢	▢	▢
Orthotic and active ROM	▢	▢	▢	▢	▢
Orthotic and Dart Throwing Motion (DTM)	▢	▢	▢	▢	▢
Active range of movement (AROM) exercises	▢	▢	▢	▢	▢
Passive range of movement (PROM)	▢	▢	▢	▢	▢

exercises					
DTM range of movement exercises	☐	☐	☐	☐	☐
Oedema management	☐	☐	☐	☐	☐
Activity advice & education	☐	☐	☐	☐	☐
Joint sense position / kinaesthesia / Mirror therapy and/or laterality training	☐	☐	☐	☐	☐
Global hand/wrist isotonic (concentric and /or eccentric) resistance training	☐	☐	☐	☐	☐
Isometric training of abductor pollicis longus (APL), flexor carpi radialis (FCR), extensor carpi radialis longus (ECRL)	☐	☐	☐	☐	☐
Isokinetic training	☐	☐	☐	☐	☐
Co-activation exercises	☐	☐	☐	☐	☐
Whole body conditioning and/or Kinetic chain optimisation	☐	☐	☐	☐	☐
Perturbation training via progressive weight-bearing exercises	☐	☐	☐	☐	☐
Perturbatio*n*	☐	☐	☐	☐	☐

training via Gyroscope and/or rhythmic stabilisation and/or Slosh pipe training					
Plyometric training	☐	☐	☐	☐	☐
Sport or work specific functional exercises	☐	☐	☐	☐	☐

19. Are there any other treatment strategies (that are not listed above) that you would implement with this patient vignette **in weeks 7 to 11 post injury?**

☐ Yes
☐ No

20. What are the other treatment strategies (that are not listed above) that you would implement with this patient vignette **in weeks 7 to 11 post injury?**

____

Section 6 - Rehabilitation 12 weeks onwards post injury.

Using the information available in the vignette as a reference, **please consider treatment strategies you would use for this patient in week 12 onwards post injury.**

A copy of the vignette is available to download, if required, on the below link.

https://acrobat.adobe.com/link/track?uri=urn:aaid:scds:US:176cd50c-9972-3cdd-9de5-02375d3cfbda

Glossary of terms, if required, is available at the link below:

https://acrobat.adobe.com/link/track?uri=urn:aaid:scds:US:de8116eb-e4ea-31af-99b2-b79487e0c03b

21. **Rehabilitation 12 weeks onwards post injury:** What rehabilitation or management strategies would you recommend for this patient in **week 12 onwards post injury??**

	Never	Rather infrequently	Some of the time	Quite often	Always
Immobilisation in POP	☐	☐	☐	☐	☐

Immobilisation in Orthotic (splint)	☐	☐	☐	☐	☐
Orthotic and active ROM	☐	☐	☐	☐	☐
Orthotic and Dart Throwing Motion (DTM)	☐	☐	☐	☐	☐
Active range of movement (AROM) exercises	☐	☐	☐	☐	☐
Passive range of movement (PROM) exercises	☐	☐	☐	☐	☐
DTM range of movement exercises	☐	☐	☐	☐	☐
Oedema management	☐	☐	☐	☐	☐
Activity advice & education	☐	☐	☐	☐	☐
Joint sense position / kinaesthesia / Mirror therapy and/or laterality training	☐	☐	☐	☐	☐
Global hand/wrist isotonic (concentric and /or eccentric) resistance training	☐	☐	☐	☐	☐
Isometric training of abductor pollicis longus (APL), flexor carpi radialis (FCR), extensor carpi radialis longus (ECRL)	☐	☐	☐	☐	☐
Isokinetic	☐	☐	☐	☐	☐

training					
Co-activation exercises	☐	☐	☐	☐	☐
Whole body conditioning and/or Kinetic chain optimisation	☐	☐	☐	☐	☐
Perturbation training via progressive weight-bearing exercises	☐	☐	☐	☐	☐
Perturbatio*n* training via Gyroscope and/or rhythmic stabilisation and/or Slosh pipe training	☐	☐	☐	☐	☐
Plyometric training	☐	☐	☐	☐	☐
Sport or work specific functional exercises	☐	☐	☐	☐	☐

22. Are there any other treatment strategies (that are not listed above) that you would implement with this patient vignette at **12 weeks onwards post injury?**

    ☐ Yes
    ☐ No

23. What are the other treatment strategies (that are not listed above) that you would implement with this patient vignette at **12 weeks onwards post injury?**

    ____

Section 7 – Measuring Effectiveness

In this section, please indicate the measures you would use to evaluate effectiveness of treatment for patient in the vignette.

A copy of the vignette is available to download, if required, on the below link.

https://acrobat.adobe.com/link/track?uri=urn:aaid:scds:US:176cd50c-9972-3cdd-

9de5-02375d3cfbda

Glossary of terms, if required, is available at the link below:

https://acrobat.adobe.com/link/track?uri=urn:aaid:scds:US:de8116eb-e4ea-31af-99b2-b79487e0c03b

24. Consider how you evaluate the effectiveness of your management strategies? Include ALL the subjective and objective measures, patient reported outcome measures (PROMs) & patient reported experience measures (PREMs) you use with this patient vignette **in weeks 3-6 post injury?**

- ☐ Joint position sense
- ☐ Range of motion
- ☐ Maximum grip strength / Grip strength ratio / Limb symmetry
- ☐ Pain free grip
- ☐ Exercise progression
- ☐ Pain scales e.g., Visual analogue scale (VAS), Numerical rating scale (NRS), Verbal rating scale (VRS)
- ☐ Disabilities of the Arm, Shoulder and Hand (DASH) questionnaire
- ☐ Quick-Disabilities of the Arm, Shoulder and Hand (Quick-DASH) questionnaire
- ☐ Patient-Rated Wrist Evaluation (PRWE)
- ☐ Euro-Qol five dimensions questionnaire (EQ-5D)
- ☐ Musculoskeletal Health Questionnaire (MSK-HQ)
- ☐ Patient satisfaction

25. Consider how you evaluate the effectiveness of your management strategies? Include ALL the subjective and objective measures, patient reported outcome measures (PROMs) & patient reported experience measures (PREMs) you use with this patient vignette **in weeks 7-11 post injury?**

- ☐ Joint position sense
- ☐ Range of motion
- ☐ Maximum grip strength / Grip strength ratio / Limb symmetry
- ☐ Pain free grip
- ☐ Exercise progression
- ☐ Pain scales e.g., Visual analogue scale (VAS), Numerical rating scale (NRS), Verbal rating scale (VRS)
- ☐ Disabilities of the Arm, Shoulder and Hand (DASH) questionnaire
- ☐ Quick-Disabilities of the Arm, Shoulder and Hand (Quick-DASH) questionnaire
- ☐ Patient-Rated Wrist Evaluation (PRWE)
- ☐ Euro-Qol five dimensions questionnaire (EQ-5D)

- ▢ Musculoskeletal Health Questionnaire (MSK-HQ)
- ▢ Patient satisfaction

26. Consider how you evaluate the effectiveness of your management strategies? Include ALL the subjective and objective measures, patient reported outcome measures (PROMs) & patient reported experience measures (PREMs) you use with this patient vignette **at 12 weeks onwards post injury?**

- ▢ Joint position sense
- ▢ Range of motion
- ▢ Maximum grip strength / Grip strength ratio / Limb symmetry
- ▢ Pain free grip
- ▢ Exercise progression
- ▢ Pain scales e.g., Visual analogue scale (VAS), Numerical rating scale (NRS), Verbal rating scale (VRS)
- ▢ Disabilities of the Arm, Shoulder and Hand (DASH) questionnaire
- ▢ Quick-Disabilities of the Arm, Shoulder and Hand (Quick-DASH) questionnaire
- ▢ Patient-Rated Wrist Evaluation (PRWE)
- ▢ Euro-Qol five dimensions questionnaire (EQ-5D)
- ▢ Musculoskeletal Health Questionnaire (MSK-HQ)
- ▢ Patient satisfaction

27. Are there any other strategies (that are not listed in the above questions), that you use to evaluate treatment effectiveness with this patient vignette?

- ▢ Yes
- ▢ No

28. Please state the other strategies you use to evaluate treatment effectiveness with patient vignette and the time points you use them e.g. 3-6 weeks, 7-11 weeks or 12 weeks onwards post injury

- ____

Section 8 – Withdrawal from study

Thank you for considering participating in this survey. Unfortunately, due to not meeting the inclusion criteria you will now exit the survey.

If you have any queries or concerns about this survey, please contact Mr. Martin Holmes (mkh388@student.bham.ac.uk) - MSc Student, University of Birmingham.

Please exit the survey by pressing the submit button.

Section 9 - Completion and Survey Submission

Thank you for completing this online survey investigating the rehabilitation practices of UK therapists in the conservative treatment of stage-one scapholunate instability.

By submitting the survey, I confirm that;

- I have answered this survey of my own free will
- **I have only completed the survey once**
- I have checked my answers and they reflect my current practice
- I understand that as the data is anonymised at the point of submission, I will not be able to withdraw my responses once I submit the survey.

If you require any further information, please contact primary investigator, Mr. Martin Holmes (MSc Student, University of Birmingham) via mkh388@student.bham.ac.uk, or study supervisor Mr. Michael Mansfield via m.mansfield@bham.ac.uk

Supplementary file D – Free text answers

**Other treatment strategies responses**	
3-6 weeks	"Allow the patient to continue to perform strengthening on contra lateral hand and lower body (e.g., don't stop cardio exercise or strength training) but advice +++"
	"Active assisted wrist Rom exercises for range & proprio feedback"
	"General health advice, optimisation of diet, general health advice re smoking, drinking etc."
	"Taping - we are missing information on the assessment to see if the joint is stiff, inflamed or hyper mobile and the risk vs benefit of moving early. "
	"At this stage I would start isometric of Brachioradialis and when this becomes stable also start ECU isometric exs and lots of y rehab "
	"Shoulder/ Elbow / DRUJ AROM"
	"Proprioception exercises, gentle ball rolling on table (seated), wrist stability exercise with lever pronation/supination"
	"Taping techniques"
6-11 weeks	"Flexor carpi radialis firing exercise"
	"Working on movement pattern - often disturbed - I would assume this wrist to be stiff rather than hyper or unstable - patient tends to a overused wrist radial extension"
	"At this stage I would start full weight bearing exs if pt is pain free. I will plan also would recommend Pilates exs"
12+ weeks	"Forearm muscle strengthening"
	"If there was intrusive pain then consider corticosteroid injection"
	"Ongoing self-management strategies, pain diary if needed."
	"Same as before. (Working on movement pattern - often disturbed - I would assume this wrist to be stiff rather than hyper or unstable - patient tends to a overuse wrist radial extension "
**Other evaluation strategy responses**	
3-6 weeks	"Patient specific functional scale at all stages."
7-11 weeks	"Weight bearing on scales after 9 weeks or when starting functional weight bearing activities".
	"Patient specific functional scale at all stages."
	"Grip strength in mid prone full pronation and full supination. After 6 weeks I also measure wearing bearing using Jamar and scales."
	"7-12 weeks if they have good ROM with minimal pain, I would mostly use self-report of return to work and ADL rather than questionnaires due to time constraints in clinic."
12+ weeks	"Patient specific functional scale at all stages."
No timescale stated	"Weight bearing ability on scale- in neutral wrist and wrist extension."
	"Weight bearing tolerance on weighing scales".

	"Maximum weight transfer through the hand compared to average normal values and the other non-affected hand."
	"Use PSFS to grade return to normal. Activities and sport for patients."
	"Oxford forearm muscle strength testing."
	"Patient specific functional score to relate specific functional demands to symptoms."
	"I ask the patient to set specific functional aims at the start of treatment & evaluate whether the patient has achieved these at the end of treatment or by specific timescales or not."
	"PSFS - patient specific functional scale. Return to work. Return to sport"
	"Video pattern of movement and control at task using resistance. ability to weight bear - using a scale when this is problematic asking patient to quote wrist function on 100%, asking patient about clicking, clunking.<br>Pain: intensity rarely informs my treatment. I use intensity to see if there is a change at rest if I think there is inflammation. Pattern, duration, type, agg and ease helps me to choose and progress treatment modalities. Joint position is not giving much love in clinic with our traditional means."
	"Weightbearing tolerance, return to sport/gym activities, PSFS."
	"Weight bearing tolerance with scales or Jamar."
	"Return to work dates."
